# Direct and Indirect Effects of Serum Uric Acid on Blood Sugar Levels in Patients with Prediabetes: A Mediation Analysis

**DOI:** 10.1155/2017/6830671

**Published:** 2017-10-29

**Authors:** Thunyarat Anothaisintawee, Dumrongrat Lertrattananon, Sangsulee Thamakaison, Sirimon Reutrakul, Boonsong Ongphiphadhanakul, Ammarin Thakkinstian

**Affiliations:** ^1^Department of Family Medicine, Faculty of Medicine, Ramathibodi Hospital, Mahdiol University, Bangkok, Thailand; ^2^Division of Endocrinology and Metabolism, Department of Medicine, Faculty of Medicine Ramathibodi Hospital, Mahidol University, Bangkok, Thailand; ^3^Division of Endocrinology, Diabetes and Metabolism, Department of Medicine, University of Illinois at Chicago, Chicago, IL, USA; ^4^Section for Clinical Epidemiology and Biostatistics, Faculty of Medicine Ramathibodi Hospital, Mahidol University, Bangkok, Thailand

## Abstract

**Purpose:**

To estimate both direct and indirect effects (through obesity) of serum uric acid (SUA) on blood sugar in prediabetes patients.

**Methods:**

Prediabetes patients who came to the outpatient clinic of the Department of Family Medicine, Ramathibodi Hospital, were included in this cross-sectional study. Mediation analysis was applied to determine direct and indirect effects of SUA on glycemic parameters (fasting plasma glucose (FPG) and HbA1c) through waist circumference (WC). A mediation effect was estimated using the product-of-coefficient method with a bootstrap analysis of 1000 replications.

**Results:**

1043 patients were eligible for this study. Every 1 mg/dl increase in SUA was associated with an increase in WC and then was significantly associated with an increase in FPG by 0.082 mg/dl (95% CI: 0.010, 0.154). SUA was directly associated with FPG with a coefficient of 0.413 (95% CI: 0.049, 0.776). For HbA1c, every one mg/dl increase in SUA was associated with an increase in HbA1c level through WC by 0.006 (95% CI: 0.003, 0.010). However, SUA was not directly associated with HbA1c.

**Conclusions:**

We found that an increase in SUA was directly and indirectly associated with increased FPG but the effect of SUA on HbA1c was shown when it was mediated through WC.

## 1. Introduction

Type 2 diabetes mellitus (T2DM) is a significant public health burden for both developed and developing countries [1]. In Thailand, the prevalence of T2DM among Thai adults has rapidly risen from 2.3% in 1991 to 7.7% in 2009 [2]. Patients with prediabetes, an intermediate stage between normal glucose and overt T2DM, are at a significantly higher risk to develop overt diabetes than those with normoglycemia [3]. Therefore, identifying and modifying risk factors for diabetes should be beneficial in preventing the disease in these high-risk patients.

Many risk factors of T2DM have been identified, and one of them was serum uric acid, which is the end product of purine metabolism. It is an antioxidant in an extracellular environment, but it can induce oxidative stress in vascular smooth muscle cells, islet cells, and adipocytes [4, 5]. Intracellular oxidative stress in these cells leads to insulin resistance, one of the main pathologies in T2DM. The findings from these in vitro studies correspond to evidences from associated studies between serum uric acid and the development of T2DM in human. Previous systematic reviews and meta-analyses also showed a significant association between hyperuricemia and increased risk of T2DM [6, 7].

Serum uric acid is also a risk factor of obesity [8, 9], likely through stimulation of hepatic lipogenesis [10] and induction of oxidative stress in the mitochondria. These lead to fat synthesis and accumulation in the hepatocytes [11]. It is known that obesity, especially central obesity, is strongly associated with both insulin resistance [12–14] and T2DM [15–17]. It is possible that uric acid itself increases blood sugar levels or exerts an indirect effect through obesity; however, this has not been explored. This cross-sectional study was therefore conducted, with the aim to estimate both direct and indirect effects (through central obesity) of serum uric acid on fasting plasma glucose (FPG) and hemoglobin A1c (HbA1c) level in prediabetes patients using a mediation analysis approach. We hypothesized that uric acid itself directly increases blood sugar level by inducing insulin resistance and indirectly affects blood sugar level through obesity. The findings from this study will be useful for the understanding of the mechanism which underlies the relationship between serum uric acid and glycemic parameters. In addition, the results will be helpful in risk factor modification and prevention of T2DM in prediabetes patients, who are greatly predisposed to overt diabetes.

## 2. Methods

The design of the study was cross-sectional which utilized the baseline data of a prediabetes cohort conducted at the outpatient clinic of the Department of Family Medicine, Ramathibodi Hospital, Thailand. Adult patients with prediabetes defined as FPG ranging from 100 to 125 mg/dl or HbA1c ranging from 5.70 to 6.49% [18] were consecutively recruited during October 2014 to March 2016. These prediabetes subjects were included in the study if waist circumference and serum uric acid were measured at baseline. Patients diagnosed with diabetes at baseline were excluded. The study protocol was approved by the Ethical Clearance Committee, Faculty of Medicine, Ramathibodi Hospital, Mahidol University. All participants gave written informed consents.

### 2.1. Data Collection

Demographic data (i.e., age, sex, marital status, and educational level), family history of diabetes, and risk behaviors (i.e., current and past smoking and alcohol use) were collected by trained interviewers. Date of diagnosis of prediabetes, diagnoses of underlying diseases (i.e., hypertension, dyslipidemia, and chronic kidney disease (CKD)), and physical examinations (i.e., height and body weight) were extracted from medical records by three investigators (TA, ST, and DL). Body mass index (BMI) was calculated by dividing weight in kilogram with height in meter square. Waist circumference (WC) in centimeter (cm) was measured at the middle point between the lowest rib and iliac crest in the standing position by the trained staff.

The most recent laboratory values (i.e., FPG, HbA1c, and serum uric acid) were retrieved from laboratory databases of Medical Statistic Unit, Ramathibodi Hospital. A time lag between the date of performing these lab tests and the date of interview was less than one year. FPG and HbA1c were measured using hexokinase glucose-6 phosphate dehydrogenase and turbid metric inhibition immunoassay, respectively. The HbA1c assay at Ramathibodi Hospital has been National Glycohemoglobin Standardization Program (NGSP) certified. Serum uric acid was measured using the uricase method.

### 2.2. Statistical Analysis

Baseline characteristics of participants were presented as mean and standard deviation (SD) for continuous data and as frequency and percentage for categorical data. We used WC instead of BMI as the marker of obesity because WC was more correlated with serum uric acid than BMI in our data (*β*-coefficient for WC = 1.89, *β*-coefficient for BMI = 0.40). Moreover, several evidence suggested that WC could better predict the risk of metabolic syndrome than BMI [19–21].

Mediation analysis was applied to determine direct and indirect effects of serum uric acid on glycemic parameters (FPG and HbA1c) through WC using the two following pathways.

Path a:

(1) $$\begin{array}{c} {WC}_{i}=a_{0}+a_{1}{UA}_{i}+\sum_{k}e_{k}z_{k}. \end{array}$$

Path b:

(2) $$\begin{array}{c} \frac{FPG}{HbA1c_{i}}=b_{0}+b_{1j}{WC}_{j}+c^{\prime }{UA}_{i}+\sum_{l}e_{k}z_{k},\;z_{k}=confounders. \end{array}$$

Uric acid, WC, and glycemic parameters (i.e., FPG/HbA1c) were considered as independent, mediator, and dependent variables, respectively. Uric acid was firstly fitted on WC. Then, the second equation was constructed by fitting WC and uric acid on FPG/HbA1c. Covariables including age, sex, and history of dyslipidemia were included and adjusted in both paths, whereas smoking was adjusted in only the first path.

A mediation effect was then estimated using the product-of-coefficient method [22, 23]. A bootstrap analysis with 1000 replications was then applied to estimate average mediation effects [24]. The mediation effect was estimated for each bootstrap, averaged across 1000 replications, and its corresponding 95% confidence interval (CI) was determined using a bias-corrected bootstrap technique.

All analyses were performed using STATA version 14. *P* value less than 0.05 was the level of statistical significance for all analyses.

## 3. Results

A total of 1633 prediabetes patients were included in the prediabetes cohort, of which 1043 (63.8%) patients were assessed for WC and serum uric acid at baseline and thus they were eligible for this study. The characteristics between eligible and ineligible patients were not significantly different (see Supplementary Table 1 available online at https://doi.org/10.1155/2017/6830671). Characteristics of eligible patients are presented in Table 1. Among eligible patients, mean age was 62.76 (±8.9) years, the majority was female (62%), and smoking and alcohol use were 5% and 23%, respectively. Forty-one percent of the study's participants had family a history of diabetes mellitus in the first degree relatives. Most patients (91%) had dyslipidemia, and more than half (68%) had hypertension, but only 5% had CKD that was defined as estimated glomerular filtration rate less than 60 ml/min/1.73m^2^. Mean BMI and WC were 25.96 (±4.0) kg/m^2^ and 90.17 (±10.1) cm, respectively. Mean FPG, HbA1c, and serum uric acid were 105.44 (±8.0) mg/dl, 5.80 (±0.4)%, and 5.79 (±1.5) mg/dl, respectively. Approximately 10% of the study's participants took uric-lowering drugs.

### 3.1. Uric Acid, Waist Circumference, and FPG

**Figure 1:**
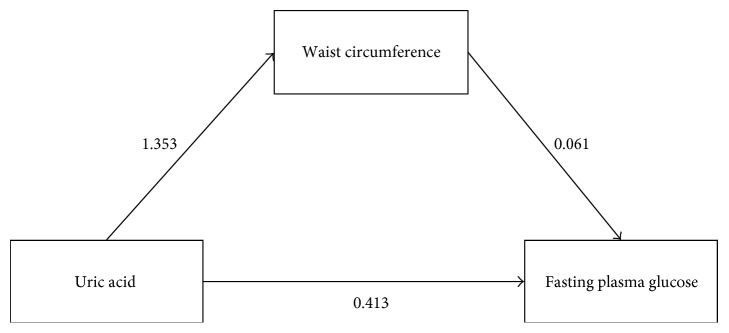
Potential causal pathway among uric acid, waist circumference, and fasting plasma glucose.

Mediation analysis was performed based on a potential causal pathway as displayed in Figure 1. Two equations, that is, mediator and outcome, were simultaneously constructed. Their coefficients adjusted for covariables were presented in Supplementary Table 2. The results from the equation of the WC mediator suggested that every 1 mg/dl increase in uric acid would be significantly associated with an increase in WC by 1.35 cm (95% CI: 0.92, 1.78). For the equation of FPG outcome, WC was significantly associated with FPG with a coefficient of 0.061 (95% CI: 0.011, 0.110). The potential causal effect of uric acid on FPG mediated by WC was then estimated by a bootstrap with 1000 replications, and the results are presented in Table 2. Every one mg/dl increase in uric acid would be associated with an increase in WC which was then significantly associated with an increase in FPG by 0.082 mg/dl (95% CI: 0.010, 0.154). In addition, uric acid was also directly associated with FPG with a coefficient of 0.413 (95% CI: 0.049, 0.776).

### 3.2. Uric Acid, Waist Circumference, and HbA1c

**Figure 2:**
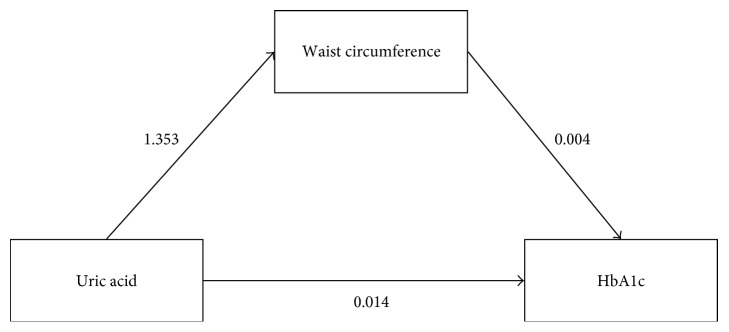
Potential causal pathway among uric acid, waist circumference, and HbA1c.

A mediation analysis of uric acid, WC, and HbA1c was performed according to a causal diagram in Figure 2. Equations for the WC mediator and HbA1c outcome adjusted for covariables were constructed (see Supplementary Table 3). The equation for the WC mediator indicated that for every one mg/dl increase in uric acid would be significantly associated with an increase in WC by 1.353 (95% CI: 0.924, 1.782). The outcome equation was also suggested that WC significantly associated with HbA1c (coefficient = 0.004; 95% CI: 0.002, 0.007). Results from 1000 replication bootstrap showed that every one mg/dl increase in uric acid would be associated with an increase in HbA1c level through WC by 0.006 (95% CI: 0.003, 0.010). However, uric acid was not directly associated with HbA1c (coefficient = 0.014; 95% CI: −0.003, 0.030) (see Table 2).

## 4. Discussion

In this study, we explored the relationship between serum uric acid and markers of glucose metabolism in patients with prediabetes and the role of WC in this relationship. The results from mediation analysis suggested that uric acid was significantly associated with FPG; that is, increasing uric acid would increase fasting plasma glucose. These effects of uric acid could be directly and indirectly mediated through WC. However, the indirect effect was minimal, when compared with the direct effect. Therefore, the effect of uric acid on FPG was mainly explained by the direct effect. In addition, uric acid was also significantly associated with HbA1c only through waist circumference. These results suggested that lowering serum uric acid could improve glycemic parameters and might decrease the risk of T2DM in prediabetes patients.

Previous evidences showed that hyperuricemia was associated with the development of T2DM [25–27]. These evidences were also replicated by a meta-analysis suggesting that each one mg/dl increase in uric acid resulted in a 17% increase risk of T2DM [6]. Moreover, in subjects with impaired glucose tolerance, serum uric acid was also significantly correlated with fasting and 2-hour plasma glucose [28]. Insulin resistance has been proposed to be the mechanism that underlies the association between serum uric acid and risk of T2DM. Uric acid produces oxidative stress via activation of nicotinamide adenine dinucleotide phosphate (NADPH) oxidase and generating oxidized lipids and inflammatory mediators [5, 29]. This oxidative stress leads to inhibition of adiponectin synthesis and results in insulin resistance. In addition, uric acid decreases endothelial nitric oxide that causes vasodilatation and facilitates delivery of glucose to the skeletal muscle [30]. Furthermore, uric acid can induce oxidative stress in islet cell, inhibiting pancreatic beta cell function [31]. In vitro study found that isolated pancreatic islets under high uric acid condition decreased basal and glucose-induced insulin secretion [32, 33]. Moreover, high serum uric acid level significantly decreased adenosine monophosphate-activated protein kinase activity, leading to an increase in hepatic glucose production [34]. These effects on inhibition of beta cell function and increase in hepatic glucose production may explain the direct effect of uric acid on blood sugar level that was found in our study.

Previous evidences also suggested that elevated serum uric acid is a risk factor of obesity [8, 9] by stimulating hepatic lipogenesis [10] and inducing oxidative stress in the mitochondria that lead to fat synthesis and fat accumulation in the hepatocytes [11]. As we know, obesity, especially central obesity, is strongly associated with insulin resistance [12–14] and T2DM [15–17]. Therefore, uric acid may induce central obesity and indirectly affect FPG through insulin resistance. This hypothesis was confirmed by our results using mediation analysis. We found that every 1 mg/dl increase in serum uric acid would result in increased WC and then would raise FPG and HbA1c level by 0.082 mg/dl and 0.006%, respectively.

However, the direct effect of uric acid was found for only FPG but not for HbA1c level. These inconsistent findings may be explained by the narrow range of HbA1c level in prediabetes population that is not varied considerably like HbA1c levels in diabetes patients. For our study's participants, HbA1c levels ranged from 4.31% to 6.49% and were quite homogeneous. In addition, study of Zhang et al. found that the association between serum uric acid and impaired fasting glucose (IFG) was different from the association between serum uric acid and prediabetes that contained HbA1c-based criteria [35]. Serum uric acid significantly increased the risk of prediabetes diagnosed by IFG and/or HbA1c criteria, while it was not significantly associated with prediabetes diagnosed by only IFG criteria. These discrepancy findings suggest that mechanisms determining the relationship between serum uric acid and FPG might be dissimilar from that between serum uric acid and HbA1c. This hypothesis should be investigated and further explored in a future study.

Our findings and previous evidence suggested the significant association between serum uric acid, blood sugar level, and risk of developing diabetes mellitus. However, causal inference of this relationship remains controversial and needs to be confirmed by the therapeutic study manipulating uric acid level by a medication or diet and its effects on blood sugar levels and risk of diabetes mellitus. Until now, there is no randomized controlled trial that investigates the benefit of serum uric acid lowering for the prevention of diabetes mellitus. However, findings from a nonrandomized study in 73 nondiabetic subjects demonstrated that participants receiving allopurinol for 3 months had significantly lower FPG, fasting insulin, and homeostatic model assessment of insulin resistance (HOMA-IR) than participants in the control group [36]. Moreover, results from a randomized open controlled trial in 176 T2DM patients also showed the significant decrease in HOMA-IR in patients receiving allopurinol for 3 years, when compared with the control group [37]. The findings from these experimental studies suggest the potential benefit of lowering uric acid level for the prevention of diabetes mellitus. However, we still need further clinical trials to prove this benefit in prediabetes subjects.

### 4.1. Strength and Limitation

To the best of our knowledge, this is the first study that assessed the direct and indirect (mediated by central obesity) effects of serum uric acid on FPG and HbA1c in prediabetes subjects. Mediation analysis was performed to assess the potential causal effects of uric acid on FPG/HbA1c using a large sample size. However, some limitations could be not avoided. Firstly, as the possible mechanism that underlies the relationship between serum uric acid and blood sugar level is insulin resistance, we did not measure fasting plasma insulin to estimate the level of insulin resistance. In addition, we did not measure 2-hour plasma glucose (by an oral glucose tolerance test) which is one of the prediabetes criteria [18]. Therefore, the relationship between serum uric acid and postprandial plasma glucose could not be explored in our study. The effect of serum uric acid on blood sugar level found in our study was marginal as one mg/dl increase in uric acid directly and indirectly raised FPG by 0.413 and 0.082 mg/dl, respectively. These effects might not be of clinical significance in routine clinical practice. Moreover, our study measured serum uric acid only one time at baseline but serum uric acid is known to be unstable and can vary overtime. Therefore, considering serum uric acid as time-varying covariate may be more accurate for assessing the relationship between serum uric acid and blood sugar level. Lastly, our study used cross-sectional data with surrogate outcomes of interest; a cohort study with incidence of T2DM should be further investigated.

In conclusion, the results from our study suggested that an increase in serum uric acid level was associated with increased FPG by both direct and indirect effects through WC. However, the associations between serum uric acid and FPG were mainly explained by the direct effect of uric acid on FPG. In addition, serum uric acid did not have a direct effect on HbA1c. The effect of uric acid on HbA1c level was shown only when it was mediated through waist circumference.

## Supplementary Material

Supplementary Table 1. Characteristics between eligible and non-eligible patients. Supplementary Table 2. Mediation analysis of uric acid, waist circumference, and fasting plasma glucose. Supplementary Table 3. Mediation analysis of uric acid, waist circumference, and HbA1c.

## Figures and Tables

**Table 1:** Characteristics of study's participants.

Characteristics	Number (%)	Mean (SD)
Age, years	—	62.76 (8.92)
Male	400 (38.35)	—
Educational level		
Primary school or lower	362 (34.77)	—
Secondary school	295 (28.34)	—
College or higher	384 (36.89)	—
Ever smoker	51 (4.89)	—
Current/past alcohol use	232 (22.26)	—
Family history of diabetes	422 (40.50)	—
Underlying diseases		
Hypertension	714 (68.72)	—
Dyslipidemia	950 (91.35)	—
Chronic kidney disease	52 (5.01)	—
Patients taking uric-lowering drugs	102 (9.78)	—
Body mass index, kg/m^2^	—	25.96 (3.95)
Waist circumference, cm	—	90.17 (10.14)
Laboratory level		
Fasting plasma glucose	—	105.44 (7.96)
HbA1c, %	—	5.80 (0.36)
Serum uric acid, mg/dl	—	5.79 (1.46)

SD: standard deviation.

**Table 2:** Causal effects of uric acid, waist circumference, and glycemic parameters.

Effects	Pathway	*β*	SE	*Z*	*P* value	Bias	95% CI
*Fasting plasma glucose*							
Indirect	➔➔UA WC FPG	0.082	0.037	2.23	0.026	−0.002	0.010, 0.154
Direct	➔UA FPG	0.413	0.185	2.23	0.026	0.005	0.049, 0.776
*HbA1c*							
Indirect	➔➔UA WC HbA1c	0.006	0.002	3.43	0.001	−0.0002	0.003, 0.010
Direct	➔UA HbA1c	0.014	0.009	1.59	0.111	−0.0003	−0.003, 0.030

CI: confidence interval; FPG: fasting plasma glucose; SE: standard error; UA: serum uric acid; WC: waist circumference.
