# Supplementary material for: Direct and Indirect Effects of Serum Uric Acid on Blood Sugar Levels in Patients with Prediabetes: A Mediation Analysis
Source: J Diabetes Res. 2017 Oct 29;2017:6830671. doi: 10.1155/2017/6830671 (PMC5682093; doi:10.1155/2017/6830671)
Supplement: Supplementary file 1 — Supplementary Table 1. Characteristics between eligible and non-eligible patients. Supplementary Table 2. Mediation analysis of uric acid, waist circumference, and fasting plasma glucose. Supplementary Table 3. Mediation analysis of uric acid, waist circumference, and HbA1c. [file 6830671.f1.docx]

**Supplementary Table 1.** Characteristics between eligible and non-eligible patients

| **Characteristics** | **Eligible**  **(n = 1066)** | **Non-eligible**  **(n = 567)** |
| --- | --- | --- |
| Age, years; mean (SD) | 62.76 (8.92) | 61.34 (8.33) |
| Male | 400 (38.35) | 163 (28.75) |
| Educational level |  |  |
| - Primary school or lower | 362 (34.77) | 198 (35.04) |
| - Secondary school | 295 (28.34) | 154 (27.26) |
| - College or higher | 384 (36.89) | 213 (37.70) |
| Ever smoker | 51 (4.89) | 23 (4.06) |
| Alcohol drinking | 232 (22.26) | 110 (19.40) |
| Family history of diabetes | 422 (40.50) | 254 (44.80) |
| Underlying diseases |  |  |
| - Hypertension | 714 (68.72) | 364 (64.42) |
| - Dyslipidemia | 950 (91.35) | 502 (88.85) |
| - Chronic kidney disease | 52 (5.01) | 13 (2.30) |
| Body mass index, kg/m^2^; mean (SD) | 25.96 (3.95) | 25.80 (4.05) |
| Laboratory level; mean (SD) |  |  |
| - Fasting plasma glucose | 105.44 (7.96) | 106.36 (7.86) |
| - HbA1c, % | 5.80 (0.36) | 5.82 (0.34) |

SD, standard deviation

**Supplementary Table 2.** Mediation analysis of uric acid, waist circumference, and fasting plasma glucose

| **Equation** | **Factors** | **β** | **SE** | **Z** | **P-value** | **95% CI** |
| --- | --- | --- | --- | --- | --- | --- |
| UA🡪WC | Uric acid | 1.353 | 0.219 | 6.18 | <0.001 | 0.924, 1.782 |
|  | Age | -0.141 | 0.034 | -4.20 | <0.001 | -0.207, -0.075 |
|  | Female | -3.562 | 0.656 | -5.43 | <0.001 | -4.847, -2.277 |
|  | Hypertension | 3.294 | 0.647 | 5.09 | <0.001 | 2.025, 4.563 |
|  | Dyslipidemia | 2.614 | 1.044 | 2.51 | 0.012 | 0.569, 4.660 |
| WC🡪FPG | WC | 0.061 | 0.025 | 2.40 | 0.016 | 0.011, 0.110 |
|  | Uric acid | 0.413 | 0.174 | 2.37 | 0.018 | 0.071, 0.755 |
|  | Smoking | 2.711 | 1.131 | 2.40 | 0.017 | 0.494, 4.927 |
|  | Family history of DM | 1.060 | 0.498 | 2.13 | 0.033 | 0.083, 2.037 |
|  | Hypertension | 1.011 | 0.533 | 1.90 | 0.058 | -0.034, 2.056 |

CI, confidence interval; FPG, fasting plasma glucose; DM, diabetes mellitus; SE, standard error; UA, serum uric acid; WC, waist circumference

**Supplementary Table 3.** Mediation analysis of uric acid, waist circumference, and HbA1c

| **Equation** | **Factors** | **β** | **SE** | **Z** | **P-value** | **95% CI** |
| --- | --- | --- | --- | --- | --- | --- |
| UA🡪WC | Uric acid | 1.353 | 0.219 | 6.18 | <0.001 | 0.924, 1.782 |
|  | Age | -0.141 | 0.034 | -4.20 | <0.001 | -0.207, -0.075 |
|  | Female | -3.562 | 0.656 | -5.43 | <0.001 | -4.847, -2.277 |
|  | Hypertension | 3.296 | 0.647 | 5.09 | <0.001 | 2.027, 4.565 |
|  | Dyslipidemia | 2.615 | 1.044 | 2.51 | 0.012 | 0.570, 4.660 |
| WC🡪HbA1c | WC | 0.004 | 0.001 | 3.87 | <0.001 | 0.002, 0.007 |
|  | Uric acid | 0.014 | 0.008 | 1.64 | 0.104 | -0.003, 0.030 |
|  | Female | 0.104 | 0.025 | 4.18 | <0.001 | 0.055, 0.153 |
|  | Family history of DM | 0.050 | 0.022 | 2.20 | 0.028 | 0.005, 0.094 |
|  | Dyslipidemia | 0.124 | 0.039 | 3.17 | 0.002 | 0.047, 0.200 |

CI, confidence interval; FPG, fasting plasma glucose; DM, diabetes mellitus; SE, standard error; UA, serum uric acid; WC, waist circumference
